# Health Care Workers’ Perspectives on Virtual Reality in Mental Health: Qualitative Interview Study

**DOI:** 10.2196/80851

**Published:** 2026-09-29

**Authors:** Anna Szczegielniak, Jakub Możaryn, Łukasz Kunert, Szymon Florek, Robert Pudlo

**Affiliations:** 1 Department of Psychoprophylaxis, Medical University of Silesia, Poniatowskiego 15, Katowice, 40-055, Poland, 48 (32) 285-43-58; 2 Institute of Automatic Control and Robotics, Warsaw University of Technology, Warsaw, Poland

**Keywords:** virtual reality, mental health, health care workers, qualitative research, technology adoption, barriers, attitudes

## Abstract

**Background:**

Virtual reality (VR) offers immersive, interactive environments, with growing evidence supporting its use in mental health diagnostics and therapy. Despite the increasing availability of VR equipment in clinical settings, integration into routine mental health care remains limited. Understanding health care workers’ attitudes, knowledge, and perceived barriers is essential for bridging the gap between technological availability and clinical implementation.

**Objective:**

This study aimed to investigate health care workers’ attitudes toward VR in mental health treatment and identify factors influencing its adoption in settings where VR equipment is already available.

**Methods:**

A qualitative study design was used, combining structured one-to-one interviews with a supporting narrative literature review. The narrative review informed the interview design and contextualized findings within the existing evidence base. Structured interviews were conducted with 16 mental health professionals (n=12, 75% women; n=4, 25% men) working in psychiatric and mental health settings in one city in southern Poland where VR equipment was available for patient care. Descriptive statistics summarized closed-ended question responses, and thematic analysis was applied to open-ended questions.

**Results:**

Of the 16 participants, 10 (62.5%) had heard of VR technology before and held a favourable view of it, but only 6 (37.5%) were aware of its applications in mental health care. Participants aged under 35 years (6/16) rated the value of VR in therapy more positively than those aged over 50 years (3/16). Among those with prior VR exposure, 85.7% (6/7) expressed confidence in its effectiveness compared with 44.4% (4/9) of those without prior exposure. The most frequently mentioned benefits included interactivity, the ability to individualize therapeutic activities to patient needs, isolated visual and auditory interactions, and an increased sense of patient involvement and control. High costs were identified as the primary barrier by 75% (12/16), and 68.8% (11/16) expressed a need for comprehensive training. Women rated their subjective VR knowledge lower than men despite demonstrating comparable objective knowledge during interviews. VR equipment was described as being restricted within certain service areas. Legal and insurance constraints limited the use of home-based VR.

**Conclusions:**

Access to VR equipment alone is insufficient to drive adoption in mental health services. Effective integration requires targeted education, comprehensive training, clear institutional guidelines on equipment responsibility and insurance, and supportive infrastructure. Addressing barriers and knowledge gaps identified in this study is essential for realizing VR’s therapeutic potential in routine clinical practice.

## Introduction

### Virtual Reality Applications in Mental Health

Virtual reality (VR) technology has evolved substantially over the past 2 decades, offering immersive, interactive environments with growing applications in mental health diagnostics and therapy [1-3]. VR enables clinicians to create controlled, reproducible therapeutic scenarios that would be difficult or impossible to replicate in traditional clinical settings. Clinical VR tools have advanced the prevention, assessment, and treatment of conditions including posttraumatic stress disorder [4], anxiety disorders [5-7], and depression [3,8].

The therapeutic potential of VR extends across a broad range of mental health conditions. VR-based interventions have demonstrated efficacy for anxiety disorders through VR exposure therapy, which allows patients to confront fear-inducing stimuli in a safe, controlled environment [6,9,10]. Meta-analyses have confirmed that VR interventions produce significant reductions in symptoms of anxiety and depression [3,8]. Beyond exposure therapy, VR has shown promise for social skill training in schizophrenia [11], pain management [12], and broader psychotherapeutic applications [13].

One factor in assessing the viability of VR therapy is treatment adherence. A meta-analysis of 46 studies on VR therapy for anxiety disorders found an overall dropout rate of 16%, lower than estimated rates for in vivo therapy and cognitive behavioral therapy (CBT) [14]. Similarly, a review of CBT in VR treatments found no significant difference in dropout likelihood between VR and traditional modalities [15], suggesting that VR-based approaches are at least as acceptable to patients as conventional treatments.

Accessibility remains a critical consideration. Digital mental health tools, including VR, must be designed to accommodate diverse populations, including those with disabilities and limited technological literacy [16,17]. The digital divide—unequal access to digital mental health resources—disproportionately affects people with serious mental illness in community settings [18]. Addressing these accessibility concerns is essential for the equitable implementation of VR in mental health services.

### Research on Health Care Workers’ Attitudes and Barriers Conducted to Date

A growing body of research has examined health care professionals’ perspectives on VR in clinical practice. Recent years have seen a substantial increase in studies investigating clinician attitudes, acceptance, and implementation barriers [19-23]. A systematic review by Bin et al [24] synthesized evidence on mental health professionals’ attitudes toward VR therapies, whereas Felnhofer et al [25] identified key barriers to adoption from the perspective of clinical psychologists and psychotherapists, including concerns about technical complexity, insufficient evidence, and lack of training. Recent reviews [26,27] of VR interventions for psychosis demonstrated the expanding scope of VR applications in severe mental illness and the corresponding need to understand clinician readiness.

Studies focused on cognitive behavioral therapists have provided important insights. Lindner et al [19] conducted a cross-sectional survey and found generally positive attitudes toward VR therapy, with therapists recognizing its potential not only for anxiety disorders but also for other conditions. However, therapists’ decisions about future VR use were more strongly influenced by negative attitudes—concerns and fears—than by positive attitudes, suggesting that addressing clinician apprehensions may be more effective than simply promoting benefits [19,28,29]. Therapists’ main concerns included doubts about the real-world transfer of therapeutic gains, worries about the quality of the virtual environment, and practical issues such as the potential impact of VR goggles on the therapeutic relationship and eye contact [20,30].

The successful integration of new technologies into health care depends substantially on the knowledge and training of health care workers. Adequate training enhances acceptance and effective use [31]. Designated champions—personnel with specific training and a clear institutional mandate—can significantly facilitate technology adoption within services [32,33]. Wray et al [20] identified that, despite the unambiguous success of VR treatments for anxiety disorders, few therapists use them in practice, pointing to a gap between evidence and implementation driven by barriers at multiple levels: individual (lack of training and unfamiliarity), institutional (cost and infrastructure), and systemic (lack of guidelines and insurance coverage).

Health care professionals’ attitudes toward adopting technological innovations are influenced by factors including perceived usefulness, ease of use, and potential to enhance patient care [34]. Positive attitudes are often linked to the technology’s perceived benefits and alignment with clinical workflows [35]. However, resistance can occur due to concerns about disrupting established practices or skepticism about effectiveness. Engaging health care workers in development and implementation processes can lead to more favorable attitudes [36]. Health care providers have reported generally favorable attitudes toward VR despite limited previous clinical use and perceived barriers to implementation [21]. Clinician-focused research further highlights the importance of practical guidance and institutional support for successful VR adoption [22].

Despite the potential benefits, several barriers hinder widespread VR adoption in mental health care. Cost is a significant factor as acquiring and maintaining VR equipment can be expensive. Safety concerns arise regarding the potential for cybersickness or exacerbation of symptoms in vulnerable populations. Ethical considerations include ensuring patient privacy, obtaining informed consent, and addressing the risk of overreliance on technology at the expense of human interaction [37,38]. Addressing these barriers requires comprehensive strategies encompassing policy development, training, and ongoing evaluation.

### Aims of This Study

The present study was conducted in mental health settings in Poland where VR equipment was already available for patient care. This unique context—investigating staff who have physical access to VR but may not use it routinely—enabled examination of whether access alone is sufficient to drive adoption. We aimed to (1) explore health care workers’ attitudes, knowledge, and perceptions regarding VR in mental health care; (2) identify barriers to and facilitators of VR adoption in settings where equipment is already available; and (3) examine how demographic factors such as age, gender, and professional experience relate to attitudes toward VR. We hypothesized that mere availability of VR equipment would be insufficient to ensure its integration into clinical practice and that training, institutional support, and prior personal experience with VR would be key factors shaping adoption.

## Methods

### Study Design

This study used a qualitative design combining structured one-to-one interviews with a supporting narrative literature review to examine health care workers’ attitudes and knowledge regarding VR in mental health care. The narrative review informed the interview design and contextualized findings within the existing evidence base. The structured interview guide was developed based on themes identified in the literature, including VR awareness, perceived benefits and barriers, training needs, and attitudes toward technology adoption in clinical settings.

### Participants

This study targeted health care workers actively engaged in mental health services. A total of 16 participants were recruited, comprising psychiatrists (n=7, 43.8%), mental health nurses (n=3, 18.8%), psychologists (n=2, 12.5%), CBT psychotherapists (n=2, 12.5%), physical therapists (n=1, 6.3%), and social workers (n=1, 6.3%). Of the 16 participants, 12 (75%) were women, and 4 (25%) were men. Most (n=11, 68.8%) had experience working only with adult patients. Participants were recruited across mental health care settings in one city in southern Poland, including a general psychiatry hospital unit, ambulatory care, and a community mental health center. All selected participants worked in services that offered patients the opportunity to participate in VR-based therapy and were involved in the care of patients taking part in a therapeutic program using VR.

Of 22 interview invitations, 6 (27.3%) individuals declined due to conflicting work commitments. The sample included individuals with diverse experience levels, from newly trained professionals to senior practitioners, to capture a range of perspectives. Demographically, efforts were made to ensure representation across gender and age groups to account for cultural and systemic influences on attitudes toward VR. .

### Data Collection

The structured interview collected responses from professionals in therapeutic fields about their experiences, perceptions, and knowledge regarding VR tools in therapy. The interview guide (Multimedia Appendix 1) covered the following domains: (1) VR familiarity—participants’ prior exposure to and subjective knowledge of VR technology, (2) perceived advantages and limitations—benefits and drawbacks of VR in therapeutic settings, (3) barriers and support needs—factors hindering VR adoption and resources required for implementation, and (4) future use—likelihood of integrating VR tools into regular clinical practice.

The interview comprised both closed-ended questions (eg, rated on Likert-type scales) and open-ended questions designed to elicit detailed perspectives. Each interview lasted approximately 20 to 30 minutes and was conducted entirely by a single researcher (AS) to ensure consistency and reduce potential interviewer bias. Interview themes and sample questions are shown in Textbox 1.

Textbox 1. **Interview questions regarding virtual reality (VR) use in clinical practice.**

**VR familiarity**

- How would you rate your knowledge of VR technology in general?
- Have you previously used VR tools in other settings (eg, entertainment, games)?
- Have you previously used VR tools in your patient care practices?

**VR in a therapeutic context**

- In your opinion, which disorder group, out of the following, could VR tools be used successfully?
- In your opinion, which disorder group, out of the following, should VR tools not be used at all?
- In your opinion, what is the biggest strength of the VR environment in a therapeutic context?

**Barriers and support needs**

- Do you have concerns regarding data security, patient privacy, and the storage of personally identifiable information within VR platforms?
- Would marketing resources or patient orientation materials be helpful in addressing potential patient resistance or reluctance to try VR?
- Would the provision of dedicated IT support personnel be a critical enabler for implementing VR in your workflow?

**Future use**

- How likely do you believe it is that VR technology will become a routinely implemented tool in your clinical environment within the next 1 to 5 years?
- How likely are you to begin using VR tools in your patient work within the next year?
- What support do you need to implement VR tools into your regular work?

### Data Analysis

With participants’ consent, face-to-face interviews were transcribed verbatim. At the beginning of each interview, participants were informed about the study’s purpose, data security measures, and their right to withdraw at any time without providing a reason. The authors identified and discussed data saturation; after reaching this point, no additional interviews were scheduled.

Data from closed-ended questions were analyzed using descriptive statistical methods to identify patterns and trends across demographic subgroups. Given the small sample size (N=16), all analyses were exploratory and descriptive; no formal inferential statistical tests were performed. Percentages are reported alongside absolute numbers throughout.

Open-ended question transcripts were analyzed using thematic analysis following the 6-phase approach described by Braun and Clarke [39]. Each transcript was carefully examined, and relevant passages were categorized during an initial, primarily deductive coding process based on the interview domains. Subcategories were then developed inductively from the data, and significant text passages were assigned to these subcategories in a second coding process.

The narrative literature review was conducted separately to inform interview design and provide context for findings. Relevant literature was sourced from PubMed, PsycInfo, and Scopus using search terms related to VR, mental health, and health care workers’ attitudes (publications up to March 2026). Inclusion criteria focused on peer-reviewed studies examining VR use in mental health care, with emphasis on adoption, clinician perspectives, and implementation barriers.

### Ethical Considerations

This study was conducted in accordance with the Declaration of Helsinki. It is part of a larger project titled “An attempt to identify factors influencing the effectiveness of physical activity in virtual reality conditions in the treatment of anxiety and depressive disorders” approved by the Bioethics Committee of the Medical University of Silesia in Katowice under the number PCN/CBN/0052/KBI/106/22. This study involved voluntary, structured interviews with health care professionals about their professional opinions on VR technology. All participants provided informed verbal consent before taking part, including consent to audio recording and transcription. Participants were assured of anonymity and informed of their right to withdraw at any time without consequence. All data were stored securely, and identifying information was removed during transcription.

## Results

### Participant Characteristics

Sixteen mental health professionals participated in the structured interviews. Table 1 summarizes participant demographics. The detailed recruitment process is presented in Figure 1.

**Table 1.** Participant demographics (N=16).

Profession	Frequency, n (%)	Gender^a^	Experience (y)^b^
Psychiatrists	7 (43.8)	5 women; 2 men	5‐25
Mental health nurses	3 (18.8)	3 women	10‐30
Psychologists	2 (12.5)	1 woman; 1 man	3‐15
CBT^c^ psychotherapists	2 (12.5)	2 women	5‐10
Physical therapists	1 (6.3)	1 woman	20
Social workers	1 (6.3)	1 man	8

a Total: 12 women; 4 men.

b Mean experience was 14.70 (SD 11.91) years.

c CBT: cognitive behavioral therapy.

**Figure 1.**
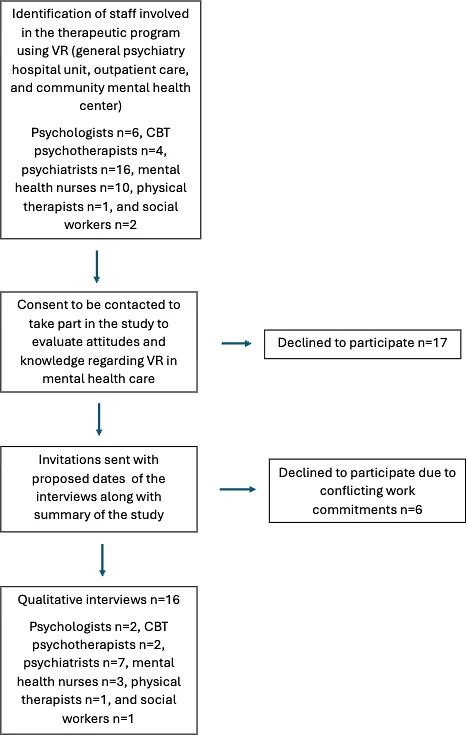
Flow diagram of participant recruitment. CBT: cognitive behavioral therapy; VR: virtual reality.

### VR Awareness and Knowledge

Participants rated their subjective VR knowledge with a mean of 2.44 (SD 1.26) out of 5 (where 1 indicated complete unfamiliarity with VR technology and 5 indicated extensive knowledge), suggesting moderate self-assessed familiarity. No clear patterns were observed between VR knowledge ratings and demographic factors such as age or years of experience.

A gender-based pattern was noted: women rated their subjective VR knowledge lower than men despite demonstrating comparable objective knowledge when asked factual questions about VR functionality during interviews. When women were asked general questions about VR equipment appearance and use (assessing objective knowledge), their responses were at the same level as those of men. This mismatch between subjective self-assessment and objective knowledge among women may reflect broader gendered patterns in technology confidence and warrants further investigation.

A positive relationship was observed between personal use of VR outside the workplace (eg, for gaming or entertainment) and self-rated VR knowledge, suggesting that personal familiarity enhances confidence (Figure 2). Higher self-rated VR knowledge was associated with a greater expressed likelihood of adopting VR tools in clinical practice, underscoring the importance of exposure and training.

One participant, a nurse aged 56 years, offered an illustrative example. Although she had never used VR herself, her son had. After hearing about his experiences, she concluded that VR could be a valuable relaxation tool for patients in a hospital ward, especially those with limited opportunities to go for walks or connect with nature. This suggests that even indirect exposure—through family members or peers—can shape positive attitudes toward VR.

Notably, mainly younger participants reported having had nonclinical contact with VR. Participants also mentioned that even when using VR outside clinical settings, it was typically done under supervision. They expressed concerns about the equipment being expensive and requiring oversight, which contributed to a feeling of limited independence with the technology. None of the 16 participants owned VR equipment for personal entertainment or gaming at home.

Overall, 87.5% (14/16) had heard about the VR project operating in their institution. However, a smaller proportion, 25.0% (4/16), had actively used VR tools in a clinical setting. These findings indicate high awareness of the internal institutional initiative, which contrasts sharply with the staff's limited pre-existing knowledge of psychiatric VR applications and their minimal hands-on clinical experience. When asked about practical applications, most respondents pointed to relaxation aspects of VR—nature scenes, breathing exercises, and guided mindfulness. In total, 31.3% (5/16) of the participants specifically mentioned exposure therapy in the context of phobias.

**Figure 2.**
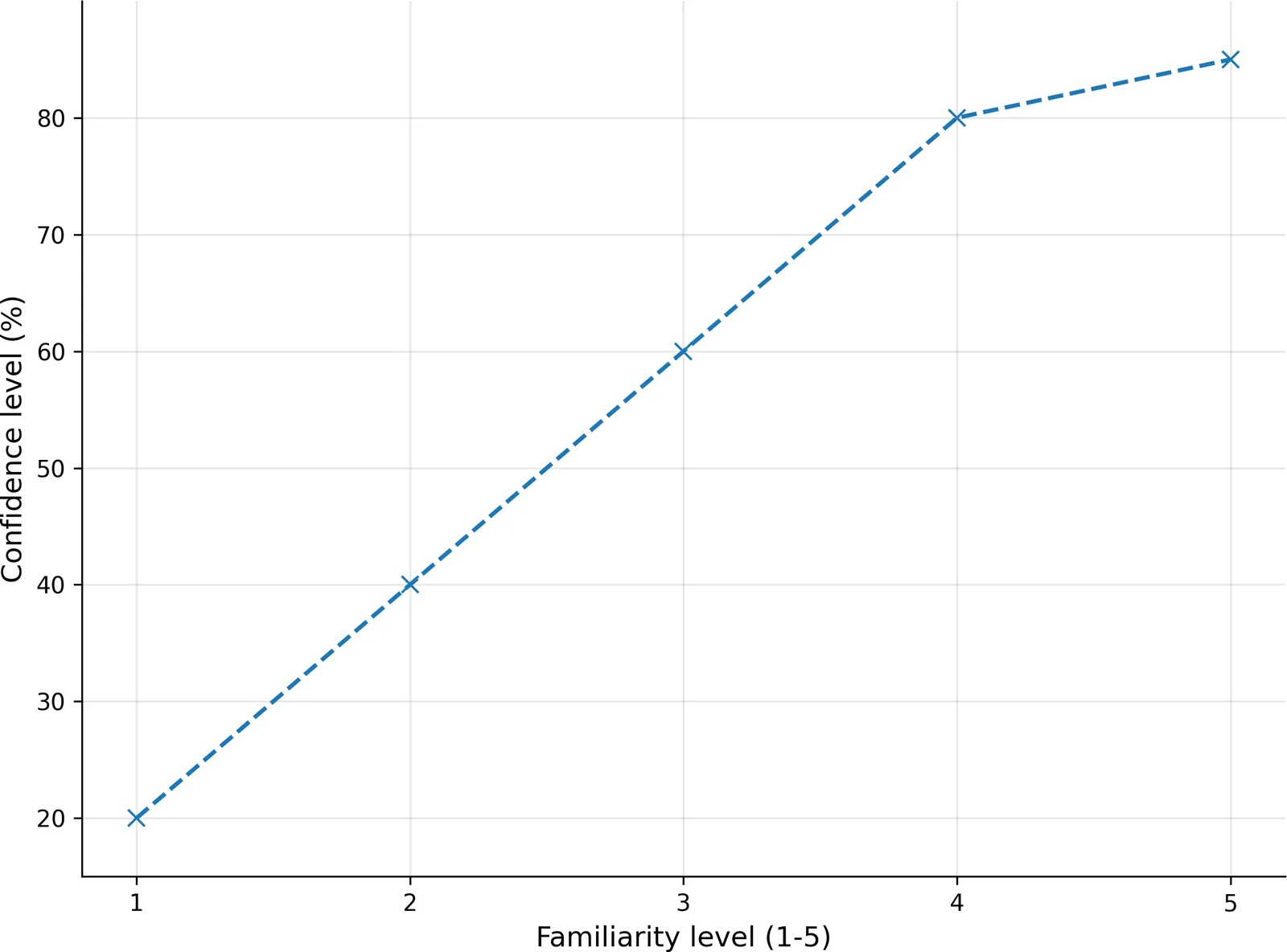
Correlation between virtual reality (VR) familiarity and confidence, emphasizing the role of exposure in shaping attitudes toward VR adoption.

### VR in the Therapeutic Context

Participants noted that VR’s ability to provide interactive, personalized, and engaging therapy was a strong motivator for its potential adoption. VR was seen as particularly promising for patient groups with conditions such as anxiety or depression but less applicable for those with severe cognitive impairments.

One participant, a psychiatrist aged 32 years, stated that nearly all patients, regardless of diagnosis, could benefit from individually tailored VR therapy. She noted the following:

My experience is that patients are eager to participate in “novelty” classes. They are drawn to technological solutions and enjoy engaging in games that can assist with cognitive rehabilitation, even if they don’t always recognize it as therapy.

Another participant, a physiotherapist aged 51 years, emphasized the importance of VR tools for individuals with psychiatric conditions:

VR can help eliminate barriers to physical activity, especially for patients with mental health disorders who often engage in low levels of physical exercise. This lack of activity can lead to chronic conditions and more pronounced side effects from medication, ultimately affecting their quality of life and increasing mortality rates. VR-based activities could provide an opportunity to integrate physical activity into their lives beyond the gym or sports, which are often inaccessible to these patients.

Among all participants, 31.3% (5/16)—3 psychiatrists, 1 psychologist, and 1 physiotherapist, all with prior VR experience—stated that no group of patients should be excluded from potential VR use. The remaining 68.8% (11/16) identified schizophrenia (during acute episodes), psychotic disorders, and advanced dementia as conditions for which VR should be avoided, citing concerns about exacerbating psychotic symptoms or causing confusion.

Practitioners expressed largely positive attitudes toward VR’s potential to enhance mental health treatment. The most frequently mentioned benefits included interactivity, the possibility of individualizing therapeutic activities according to patient needs, isolated interactions in visual and auditory perception, and an increased sense of patient involvement and control. A total of 68.8% (11/16) believed that VR could complement traditional therapies, and 62.5% (10/16) identified its adaptability as a strength.

Despite this optimism, notable knowledge gaps were evident. In total, 43.8% (7/16) of participants perceived VR primarily as a gaming tool, reflecting misconceptions about its clinical applications. A lack of understanding of VR safety protocols and evidence-based effectiveness also contributed to hesitancy among some participants.

### Barriers to VR Adoption

Several barriers to VR adoption in therapeutic settings were identified. Financial constraints emerged as the most prominent obstacle, with 75% (12/16) identifying the cost of purchasing and upgrading VR equipment as the greatest disadvantage. Notably, none of these participants could specify the actual cost of a single headset available at their institution; their estimates were, on average, €500 to €1000 higher than the actual cost (€1=US $1.15 as of August 5, 2026). This discrepancy suggests that perceptions of cost may be inflated relative to reality.

A particularly striking finding concerned the sense that VR equipment was “locked” or restricted within certain service areas. Participants reported that even when VR equipment was technically accessible, there was often no clear information regarding who was responsible in the event of damage. This ambiguity about responsibility created anxiety among staff and limited their willingness to use the equipment freely. A female psychiatrist aged 30 years highlighted a specific issue: for legal reasons surrounding equipment insurance, patients could not take VR goggles home. This limited the ability to maintain the positive therapeutic effects observed during ward-based VR sessions and reduced the likelihood of VR being incorporated into ongoing therapeutic programs.

Staff described wanting more independence with the equipment—the ability to use it during individual therapy sessions or bring it to patients’ rooms (eg, for bedridden individuals)—but felt constrained by institutional rules, unclear protocols, and insufficient guidance. Several participants noted that although written materials about the VR program were provided, participants lacked the time to seek answers independently. Joint training sessions had been limited: only 2 were held over a 6-month period during working hours, making it difficult for many staff members to participate.

Consequently, comprehensive training emerged as the secondary barrier, with 68.8% (11/16) of participants expressing a need for thorough education. Other significant obstacles included ethical and safety concerns, raised by 37.5% (6/16), such as patient privacy, risk of retraumatization, and potential overreliance on VR technology; infrastructure limitations, including limited access to dedicated space and technical support; and digital literacy concerns, noted by 25% (4/16), who observed that without proper digital education, some patients may struggle to use VR independently.

Most participants (12/16, 75%) reported that access to VR equipment at work and involvement in caring for patients in a VR-based therapeutic program had not substantially changed their attitudes toward VR. They explained that the information provided about available therapeutic options had been superficial, lacking the full range of activities offered to patients. There was no information on whether the therapeutic protocol could be modified or whether the equipment could be used in patients’ rooms or during one-on-one therapy sessions.

### Facilitators of VR Adoption

When asked about the likelihood of starting to use VR tools in patient work over the following year, participants rated their chances at a mean of 3.25 out of 5 (where 1 was “very likely” and 5 was “definitely not”). Only 6.3% (1/16) selected “very likely,” citing positive experiences during an ongoing ward-based VR therapeutic program.

Exploratory subgroup analysis indicated that participants aged under 35 years (6/16) rated the value of VR in therapy more positively than those aged over 50 years (n=3; mean 3.83, SD 0.75 vs 3.00, SD 0.00 on a 5-point scale, with higher scores reflecting more favorable attitudes). Professionals with fewer than 5 years of experience (5/16) also reported a somewhat greater likelihood of starting to use VR within the next year than those with more than 15 years of experience (6/16; mean 2.80, SD 1.10 vs 3.33, SD 1.21, respectively, with lower scores indicating greater likelihood). The latter group more often cited concerns about disrupting established practices. Among those with prior VR exposure (whether personal or professional), approximately 85.7% (6/7) expressed strong confidence in its effectiveness, compared with approximately 44.4% (4/9) of those without such exposure. This pattern underscores the potential importance of hands-on experience in shaping positive perceptions.

These subgroup patterns should be interpreted cautiously given the small sample size (N=16) and are presented as exploratory, descriptive observations rather than statistically significant differences.

In response to the question “What support do you need to implement VR tools into your regular work?” participants highlighted several priorities: support from institutional leadership, education for all department staff to encourage active participation, adequate administrative and logistical support, and high-quality visualization programs enabling immersion. A total of 18.8% (3/16) of the participants specifically indicated the need for funding covering not only a one-time equipment purchase but also the systematic development and evaluation of therapeutic programs.

A female physiotherapist aged 51 years suggested that VR-based therapeutic activities should be presented to patients as additional relaxation options rather than strict treatments, emphasizing the importance of making the experience enjoyable. Other participants recommended organizing more afternoon activities, allowing patients to engage independently without staff oversight to help them feel more comfortable. They also highlighted the need to inform patients about VR’s therapeutic mechanisms and evidence base.

## Discussion

### Principal Results

This qualitative study of 16 mental health professionals working in settings where VR equipment was available for patient care revealed a consistent pattern: physical access to VR technology alone was insufficient to drive its integration into routine clinical practice. While most participants (10/16, 62.5%) had heard of VR before the project and expressed broadly positive attitudes toward its therapeutic potential, only 37.5% (6/16) were aware of VR’s mental health applications, and only 25.0% (4/16) had actively engaged with VR in a clinical capacity. This study identified a constellation of barriers—including knowledge gaps, financial concerns, insufficient training, unclear institutional guidelines, and legal and insurance constraints—that collectively prevented adoption despite equipment availability.

Several qualitative observations were particularly noteworthy. Staff described VR equipment as being “locked” within certain areas of the service, with no clear framework for independent clinical use. Anxiety about responsibility for expensive equipment deterred staff from allowing patients to use it freely. A gender-based pattern in VR knowledge self-assessment was observed, with women rating their knowledge lower than men despite demonstrating comparable factual understanding, suggesting that confidence rather than competence may be a barrier for some professionals. Younger and less experienced professionals were descriptively more open to VR, whereas those with prior personal VR exposure—even outside clinical settings—expressed markedly greater confidence in its effectiveness.

### Comparison With Prior Research

Our findings are broadly consistent with the growing literature on clinician perspectives on VR in mental health care. Felnhofer et al [25] identified similar barriers among clinical psychologists and psychotherapists, including concerns about technical complexity, insufficient evidence, and lack of training. Lindner et al [19] found that CBT therapists held generally positive attitudes toward VR but that negative concerns—particularly about real-world transfer of therapeutic gains—more strongly predicted future use intentions than positive attitudes. This aligns with our finding that even staff with access to VR and generally favorable views hesitated to adopt it in the face of unresolved practical concerns.

The importance of prior exposure and familiarity identified in our study is consistent with findings by Wray et al [20], who noted that despite the unambiguous success of VR anxiety treatments, very few therapists use VR in practice, largely due to unfamiliarity and lack of training opportunities. Cushnan et al [22] similarly found that clinicians’ perspectives on immersive tools were shaped by hands-on experience and institutional support. Our finding that 85.7% (6/7) of participants with prior VR exposure expressed confidence in its effectiveness compared with 44.4% (4/9) without exposure reinforces the critical role of experiential learning in technology acceptance.

The misconception that VR is primarily a gaming tool, expressed by 43.8% (7/16) of our participants, is consistent with findings that clinicians often lack awareness of VR’s clinical applications [40]. At the same time, participants in our study suggested that framing VR as entertaining might paradoxically encourage patients to engage with therapeutic tools. This tension between clinical credibility and patient engagement represents an important consideration for implementation strategies.

Our study extends existing research by focusing specifically on staff working in settings where VR is already physically present but not integrated into routine practice. Previous studies have generally examined attitudes in the abstract or in settings where VR is being newly introduced. The finding that access alone is insufficient—and that institutional, legal, and training frameworks are necessary to translate access into use—adds a new dimension to the implementation literature. This is consistent with the work by Berardi et al [31], who identified systemic barriers to digital technology implementation in mental health, and by Chung et al [41], who emphasized the importance of clinician and service manager engagement.

Recent research highlights familiarity and exposure as key factors in mental health professionals’ adoption of VR. Prior experience with VR and greater knowledge of VR-based therapy have been associated with a greater likelihood of future clinical use [20,42]. Moreover, recent findings similarly indicate greater interest in therapeutic VR among professionals with prior VR experience [25,43]. Structured VR training has also been shown to improve therapists’ knowledge, skills, and confidence in using VR [43]. Furthermore, younger mental health professionals may show greater interest in therapeutic VR than older professionals. By contrast, a large recent study [25] found that years of professional experience were not significantly associated with interest in VR. In the exploratory sample under consideration, younger and less experienced professionals appeared more receptive to the use of VR. We should interpret these findings with caution, given the small subgroup sizes.

### Limitations

This study has several limitations. First, the sample of 16 participants was drawn from mental health services in a single city in southern Poland, limiting generalizability to other geographic, cultural, or health care system contexts. Second, the use of structured interviews, while ensuring consistency, may have limited the depth of qualitative exploration compared with semistructured or in-depth interview approaches. Third, the small sample size precludes formal statistical inference; all quantitative findings are descriptive and exploratory, and subgroup patterns (by age, gender, and years of experience) should not be interpreted as statistically significant differences. Fourth, potential selection bias may have influenced participation: staff with a preexisting interest in technology may have been more inclined to take part, potentially inflating positive attitudes relative to the broader workforce. Fifth, the rapidly evolving nature of VR technology means that findings may become outdated as new, more affordable and user-friendly VR systems enter the market, necessitating ongoing research. Finally, despite individual studies showing positive effects of VR therapies, there remains insufficient scientific evidence regarding the optimal use and target populations for these tools [44], which may influence clinicians’ willingness to adopt VR even when it is available.

### Conclusions

There was a broadly positive outlook on VR use in mental health care among the health care professionals interviewed in this study. However, significant barriers related to knowledge, cost, training, institutional guidelines, and legal constraints must be addressed before VR can be effectively integrated into routine clinical practice. Free access to VR equipment may foster more positive attitudes, but a lack of knowledge about how to use it—combined with anxiety about equipment responsibility and the absence of clear protocols—decreases willingness to implement VR in daily therapeutic routines and may hinder its introduction as a permanent tool in mental health services.

Financial constraints extend beyond initial equipment purchase. It is equally important to establish clear rules for equipment use, create appropriate insurance conditions, and ensure systemic access to training and evaluation of therapeutic programs. Targeted education, comprehensive training, and supportive institutional infrastructure are essential to overcome the barriers identified in this study and fully realize VR’s potential as a therapeutic tool. Future research should include larger, multisite samples; use semistructured interview methods to capture richer qualitative data; and investigate the effectiveness of specific implementation strategies—such as VR champion programs, structured training curricula, and institutional policy frameworks—in translating equipment access into routine clinical use.

A visual abstract summarizing the main study findings, implementation barriers, and integration requirements is presented in Multimedia Appendix 2.

## Supplementary material

10.2196/80851 Multimedia Appendix 1 Interview guide used in this study.

10.2196/80851 Multimedia Appendix 2 Visual Abstract summarizing health care workers' perspectives, key implementation barriers, and requirements for successful virtual reality integration in mental health care.

## Abbreviations

CBT: cognitive behavioral therapy
VR: virtual reality
