# Supplementary material for: Health Care Workers’ Perspectives on Virtual Reality in Mental Health: Qualitative Interview Study
Source: JMIR Hum Factors. 2026 Sep 29;13:e80851. doi: 10.2196/80851 (PMC13625927; doi:10.2196/80851)
Supplement: Supplementary file 1 [file humanfactors-v13-e80851-s001.docx]

**Incorporating VR Tools into Therapeutic Practice**

Objective 1: What are mental health professionals' perspectives on the benefits and drawbacks of employing virtual reality in therapy for patients with mental health disorders?

Objective 2: How can recruitment and participation in such VR-based projects be improved?

Demographic data

1. Age (in years)

2. Gender (Male/Female/Non-binary/Prefer not to say)

3. Job title/profession: psychotherapist, psychologist, psychiatrist, nurse, recovery assistant, physiotherapist; specify others:_______

4. Years of professional experience working with patients: (in years)

5. Patient groups: adults, children, adolescents, or both

6. Main disorder categories you work with (pick up to 3): dementia, addictions, schizophrenia/psychotic disorders, affective disorders, anxiety/stress-related disorders, eating disorders, sleep disorders, personality disorders; specify others:________

Structured Interview

1. How would you rate your knowledge of VR technology in general?

| 1 | 2 | 3 | 4 | 5 |
| --- | --- | --- | --- | --- |
| I don’t know anything about it |  | I’ve basic knowledge |  | I’ve an extensive knowledge of VR technology |

2. Have you previously used VR tools in other settings such as entertainment or gaming? Y/N If so, please specify where and when, along with your initial impressions and feelings.
3. Do you own VR equipment for personal entertainment or gaming at home? Y/N
4. List the standard components of VR equipment that create an immersive 3D experience.
5. What do you estimate is the average cost of basic VR gear suitable for mental health therapy purposes, like relaxation, phobia exposure, or mindfulness, per patient (in PLN)?
6. Have you previously observed VR tools in patient care? Y/N If yes, provide details about where and when, and your initial thoughts and feelings.
7. Have you previously used VR tools in your patient care practices? Y/N If so, please specify where and when, along with your initial impressions and feelings.
8. Are you aware of any current clinical research or evidence supporting VR use for the patient groups you serve? Y/N If so, please summarize or provide details.
9. Are you aware of any practical applications of VR use for the patient groups you serve? Y/N If so, please list them.
10. Do you see VR primarily as an entertainment/gaming tool or a clinical tool?
11. Has access to VR equipment at work or direct involvement in caring for patients in a VR-based therapeutic program changed their attitudes toward VR? Y/N
12. In your opinion, which disorder group, out of the following, could VR tools be used successfully: dementia, addictions, schizophrenia/psychotic disorders, affective disorders, anxiety/stress-related disorders, eating disorders, sleep disorders, personality disorders; other:________, none. Why?
13. In your opinion, which disorder group, out of the following, should VR tools not be used at all: dementia, addictions, schizophrenia/psychotic disorders, affective disorders, anxiety/stress-related disorders, eating disorders, sleep disorders, personality disorders; other:________, none. Why?
14. Do you think VR can effectively complement traditional therapies in mental health care? Y/N
15. How would you rate the overall value and utility of virtual reality technology in a mental health therapeutic context?

| 1 | 2 | 3 | 4 | 5 |
| --- | --- | --- | --- | --- |
| No value |  | Moderate value |  | Extreme value |

16. Name, in your opinion, the biggest strength of the VR environment in a therapeutic context.
17. Of the following, what do you think are the strongest motivators for adopting VR in a clinical environment? (choose up to 3) Interactivity, innovative approach, ability to individualize therapeutic activities according to patient needs, efficiency, ability to use tools outside the healthcare facility, ability to collect patient data in real time, increased sense of engagement and control in patients. Why?
18. Name, in your opinion, the biggest weakness of the VR environment in a therapeutic context.
19. Of the following, what do you think are the biggest barriers for adopting VR in a clinical environment? (choose up to 3) The cost of acquiring and upgrading VR equipment, technical difficulties in operating VR equipment, reluctance of some patients to use the new technology, patients unable to understand and use the technology on their own, side effects of use such as nausea, and a lack of sufficient scientific criteria and recommendations for the use of VR. Why?
20. What do you see as the biggest limitation of using VR in your daily work with patients in your workplace?
21. What support do you need to implement VR tools into your regular work?
22. Would the provision of dedicated IT support personnel be a critical enabler for implementing VR in your workflow? Y/N
23. Would the availability of standardized clinical guidelines and evidence-based protocols facilitate the integration of VR into your practice? Y/N
24. Would marketing resources or patient orientation materials be helpful in addressing potential patient resistance or reluctance to try VR? Y/N
25. Do you require a stronger business case demonstrating the financial viability and long-term value of VR for your specific clinical service? Y/N
26. What specific types of comprehensive training (technical and clinical) would you require to feel confident in delivering VR-supported therapies?
27. Do you have concerns regarding data security, patient privacy, and the storage of personally identifiable information within VR platforms? Y/N
28. What can you do to increase the effectiveness of patient recruitment to therapeutic programs using VR tools?
29. How likely do you believe it is that VR technology will become a routinely implemented tool in your clinical environment within the next one to five years?

| 1 | 2 | 3 | 4 | 5 |
| --- | --- | --- | --- | --- |
| Not likely at all |  | Possible |  | I’m sure it’ll happen |

30. How likely are you to begin using VR tools in your patient work within the next year?

| 1 | 2 | 3 | 4 | 5 |
| --- | --- | --- | --- | --- |
| Very likely |  | Possible |  | Definitely not |
